# Remembering the truth or falsity of advertising claims: A preregistered model-based test of three competing theoretical accounts

**DOI:** 10.3758/s13423-024-02482-8

**Published:** 2024-03-25

**Authors:** Lena Nadarevic, Raoul Bell

**Affiliations:** 1 Department of Psychology, School of Social Sciences, University of Mannheim, D-68131 Mannheim, Germany; 2 University of Applied Sciences Fresenius Heidelberg, Heidelberg, Germany; 3 Heinrich Heine University Düsseldorf, Düsseldorf, Germany

**Keywords:** Cartesian model, Spinozan model, Expectations, Advertising claims, Feedback memory, Multinomial model, Veracity feedback

## Abstract

Given the large amount of information that people process daily, it is important to understand memory for the truth and falsity of information. The most prominent theoretical models in this regard are the Cartesian model and the Spinozan model. The former assumes that both “true” and “false” tags may be added to the memory representation of encoded information; the latter assumes that only falsity is tagged. In the present work, we contrasted these two models with an expectation-violation model hypothesizing that truth or falsity tags are assigned when expectations about truth or falsity must be revised in light of new information. An interesting implication of the expectation-violation model is that a context with predominantly false information leads to the tagging of truth whereas a context with predominantly true information leads to the tagging of falsity. To test the three theoretical models against each other, veracity expectations were manipulated between participants by varying the base rates of allegedly true and false advertising claims. Memory for the veracity of these claims was assessed using a model-based analysis. To increase methodological rigor and transparency in the specification of the measurement model, we preregistered, a priori, the details of the model-based analysis test. Despite a large sample size (*N* = 208), memory for truth and falsity did not differ, regardless of the base rates of true and false claims. The results thus support the Cartesian model and provide evidence against the Spinozan model and the expectation-violation model.

With the rise of the digital age, it is easier than ever to disseminate and access information. While the widespread availability of information is often an advantage, there is also a disadvantage: the proliferation of false information (Kozyreva et al., 2020). Therefore, it becomes an even more pressing question to understand how the human mind processes and represents the veracity of information. This question was raised long before the digital age by the philosophers Descartes and Spinoza. Building on their philosophical perspectives, Gilbert and colleagues (Gilbert et al. 1990; 1993) distinguished between two concurrent cognitive models on the mental representation of truth and falsity: the *Cartesian model* and the *Spinozan model*.

## The Cartesian model and the Spinozan model

The Cartesian model (developed from the work of Descartes, 1984/1644) assumes that representations about the state of the world possess no inherent veracity. However, if a person has sufficient cognitive capacity to evaluate a piece of information or receives evidence of its truth or falsity, their memory representation is updated to include the truth status. Metaphorically speaking, the memory representation of an encoded piece of information is marked with a “true” or “false” tag (see Gilbert et al., 1990). Within memory research, the term “tag” is used to refer to contextual information that is attached to a memory representation (Bell et al., 2012; Graesser & Nakamura, 1982; Küppers & Bayen, 2014; Nadarevic & Erdfelder, 2013). For instance, a statement such as *“Wearing face masks protects against COVID-19.”* may be tagged as ”true” after learning that the claim is supported by scientific evidence. The concept of tagging implies that the veracity of a statement is not inferred directly from the content of the statement itself or a generic representation such as “all theories are false” but instead is treated as a separate representation that is assigned to the memory representation of the statement. According to the Cartesian model, representations of information in memory can therefore have three states: untagged, tagged as “true”, and tagged as “false”.

By contrast, the Spinozan model (originating from the work of Spinoza, 2006/1677) postulates that representations about the world are inherently represented as true by default, under the premise that to cognitively represent something fundamentally implies acknowledging its existence. Therefore, every piece of information stored into memory is initially considered true. However, if a person has sufficient cognitive capacity to evaluate a piece of information or encounters evidence of its falsity, the memory representation may be subsequently tagged as “false” to explicitly represent the falsity of that information. For instance, a statement such as *“Eating carrots improves your vision.”* would be accepted as true unless explicitly represented as false. Thus, according to the Spinozan model, there are only two different states of memory representations, namely untagged and tagged ones. The former are believed to be “true,” the latter are tagged as “false.”

Because the Spinozan model assumes that only falsity but not truth is tagged, the mechanism that is specified by this model can be considered more resource-efficient than that of the Cartesian model. At the same time, however, the mechanisms specified by the Spinozan model may be more fallible, because false information will be remembered as “true” if the tagging process fails. Indeed, several empirical studies found support for the prediction that distraction and shallow information processing at encoding impair memory for “false” feedback, but not memory for “true” feedback (Gilbert et al., 1990, 1993; Koslow & Beltramini, 2002). These studies advocating the Spinoza model, however, measured memory for truth and falsity at the level of directly observable behavior by assessing the proportion of (in)correct feedback attributions. This is problematic in that this measure confounds item memory (i.e., memory for the statement itself), feedback memory (i.e., memory for the veracity of the statement), and multiple guessing processes. Indeed, studies that controlled for potential guessing biases (e.g., Nadarevic & Erdfelder, 2013; 2019; Street & Kingstone, 2016; Street & Richardson, 2015) favored the Cartesian model over the Spinozan model: When memory was uncontaminated by guessing, memory for truth and falsity was found to be equally good in most experiments. In one experiment (Nadarevic & Erdfelder, 2019, Experiment 2), memory for truth was even better than memory for falsity when no distraction was present, suggesting that tagging of truth and falsity may be context-dependent.

### The expectation-violation model

The Cartesian model and the Spinozan model share a common inflexibility in that the tagging process is thought to be invariant to the situational context in which the information is processed. Therefore, a third option is considered here: a tagging process that relies upon the statistical information of the situational context. Consider, for instance, a context in which most of the information can be trusted. In such a context it is efficient to accept all incoming information unless there is reason to revise this expectation, in which case the information is tagged as “false”. However, in a context in which most of the information cannot be trusted, it may be more efficient to consider all information as false unless there is reason to revise this expectation in which case the information is tagged as “true”. This expectation-violation account originates in the schema-copy-plus tag model (Graesser & Nakamura, 1982), according to which schema-incongruent information is tagged in memory. Empirical findings suggest an even broader tagging process that marks specific instances in which more general situational models have to be revised (e.g., Bell et al., 2010; Kroneisen et al., 2015; Schaper et al., 2019). An interesting feature of the expectation-violation model is that it predicts that people may sometimes tag the falsity of information (when truth is expected), may sometimes tag both falsity and truth (when there are no expectations in either direction), and may sometimes tag the truth of information (when falsity is expected). The model is interesting to consider because it can incorporate conflicting findings on the competing models proposed by Gilbert et al. (1990). However, to our knowledge, the expectation-violation model has not yet been directly tested with respect to memory for truth and falsity – a research gap that we close with the present work.

### The present work

In the present study, the Cartesian model, the Spinozan model, and the expectation-violation model were tested against each other. Participants were presented with statements that were indicated to be true or false, or that appeared without veracity feedback. To manipulate the expectations about statement veracity, the base rates of true and false statements were manipulated between groups. In a memory test, all participants were asked to remember whether a claim was old or new. In the case of an old judgment, they were also asked to indicate whether the claim had been displayed with “true” feedback, “false” feedback, or without feedback. As in previous studies on memory for truth and falsity (Nadarevic & Erdfelder, 2013, 2019), a formal measurement model was used to differentiate between different memory processes and guessing biases. Specifically, to capture feedback memory unconfounded by item memory and guessing processes, we used the two-high-threshold variant of the three-sources model of Riefer et al. (1994), which belongs to the class of multinomial processing tree models (MPT models), for the data analyses.

To increase methodological rigor and transparency in the specification of the model, the process of selecting the model for measuring feedback memory was preregistered a priori in a predetermined sequence of steps. Furthermore, we preregistered the hypotheses that were derived from the different models and how they were to be tested against the data. According to the Cartesian model, people should tag allegedly false claims with a “false” tag and allegedly true claims with a “true” tag. Consequently, “true” and “false” feedback should be remembered equally well in the memory test, regardless of the base rates of true and false information. According to the Spinozan model, people should only tag allegedly false claims with a “false” tag whereas all other claims should remain untagged. Consequently, memory for “true” feedback should be significantly worse than memory for “false” feedback, because claims with “true” feedback should be indistinguishable from claims without veracity feedback, regardless of the base rates of true and false information. According to the expectation-violation model, people should tag information that violates their expectations in a given context. Consequently, people should show significantly better memory for “false” compared to “true” feedback in a context where most of the information that is encountered is true. In contrast, significantly better memory for “true” compared to “false” feedback should be obtained in a context where most of the information encountered is false.

## Methods

We report how we determined our sample size, all data exclusions, all manipulations, and all measures in the study. The sample size, the materials and procedure, the hypotheses, the process for selecting the measurement model, and the statistical tests were preregistered on February 13, 2023, on the Open Science Framework (OSF): https://osf.io/6hq4y.

### Participants

Data collection took place via the crowdsourcing platform *Prolific* from February 16 to 20, 2023. Only prolific workers who fulfilled the following prescreening criteria were eligible to participate: German as first language, age between 18 and 50 years, and minimum approval rate of 95%. Participants received £3.00 for completing the experiment, which took about 20 min. As preregistered, we terminated data collection when our sample included $N = 208$ eligible participants. From 209 data sets, one was excluded because the participant took notes during the experiment. The final sample (124 male, 80 female, four diverse) had a mean age of *M* = 30 (*SD* = 7) years.

### Materials

We used fictitious product claims as stimuli because such statements have some ambiguity concerning their truth status. Our stimuli came from a large set of product claims developed and pretested for credibility (-3 = *not credible at all* to +3 = *highly credible*) by Bell and colleagues (2021; 2022). All claims referred to fictitious brands, excluding any impact of brand knowledge or product experience on the results. We selected 90 claims that were hypothetically testable (e.g., *“Gaton jam consists of fruits cooked directly without additives.”*) and that were not particularly (un)credible according to the pretest norms (*M* = 0.07, *SD* = 0.64). The materials are available on the OSF: https://osf.io/n9mj7/.

### Procedure

After consenting to participate in the study, participants completed a brief demographic questionnaire. They were randomly assigned to one of two experimental groups characterized by different base rates of allegedly “true” and “false” advertising claims. In the high “true” base rate group (HTB group: *n* = 103), most of the advertising claims in the study phase were marked as “true” while in the high “false” base rate group (HFB group: *n* = 105), most of the claims were marked as “false”.

Participants of both groups were asked to imagine the hypothetical scenario that an institute for consumer protection had tested the veracity of various advertising claims. The HTB group was informed that the majority of all the advertising claims tested had turned out to be true. In contrast, the HFB group was informed that the majority of all the advertising claims tested had turned out to be false. Participants of both groups were presented with 60 advertising statements that appeared consecutively in random order. Of these, 48 statements appeared together with a badge that provided feedback on the statement’s veracity. A green badge with a thumbs-up indicated that the claim was true (“true” feedback condition). A red badge with a thumbs down indicated that the statement was false (“false” feedback condition). The remaining 12 statements were displayed without a badge indicating that the veracity of these statements has not been checked (“unchecked” feedback condition). In the HTB group, 36 statements were displayed with a “true” badge, 12 with a “false” badge, and 12 without a badge. Accordingly, in the HFB group, 36 statements were displayed with a “false” badge (and 12 each with a “true” badge or without a badge). The statements were randomly assigned to the conditions. Participants of both groups were instructed to memorize the presented information.

Following the study phase, participants were asked to respond to the skepticism-towards-advertising scale (Obermiller & Spangenberg, 1998), consisting of nine items. Finally, in a subsequent memory test, 90 advertising statements (60 old and 30 new ones) appeared in random order. For each statement, participants had to indicate whether they had seen the statement during the study phase (“old”) or not (“new”). For statements classified as “old”, participants had to additionally indicate whether the statement had been presented with a “true” badge, a “false” badge, or without a badge in the study phase (“true” vs. “false” vs. “unchecked”).

### Design

The base rate of “true” and “false” feedback was manipulated between participants (HTB group vs. HFB group) and feedback condition (“true” vs. “false” vs. “unchecked”) was manipulated within participants. Moreover, participants were assigned to four different counterbalancing conditions. These four conditions differed in the arrangement of response options in the item-memory test (“old-new” versus “new-old”) and feedback-memory test (“true-false-unchecked” or “false-true-unchecked”).

### Data analysis

Data analyses were conducted with R (R Core Team, 2022) and strictly followed the preregistered analysis plan. The multinomial processing tree analyses were conducted with the package MPTinR (Singmann & Kellen, 2013). For the preregistered secondary analyses, we used the packages afex (Singmann et al., 2023), emmeans (Lenth, 2022) and effectsize (Ben-Shachar et al., 2020). The data and the R-code for the reported analyses are available on the OSF: https://osf.io/92rxy/ (data); https://osf.io/k72w6/ (code).

We used the two-high-threshold variant of the three-sources model of Riefer et al. (1994) for data analyses. This MPT model has been successfully validated and used in prior research (Bayen et al., 1996; Bell et al., 2010; Keefe et al., 2002; Nadarevic & Erdfelder, 2013, 2019). MPT models are stochastic models for discrete data that allow to disentangle and to estimate the contribution of different latent cognitive processes underlying observable responses based on maximum likelihood estimation (for a review, see Erdfelder et al., 2009; for a tutorial, see Schmidt et al., 2023). Using the MPT model thus allowed us to disentangle, to estimate, and to compare parameters representing item memory, feedback memory, and guessing processes in our experiment.

**Fig. 1:**
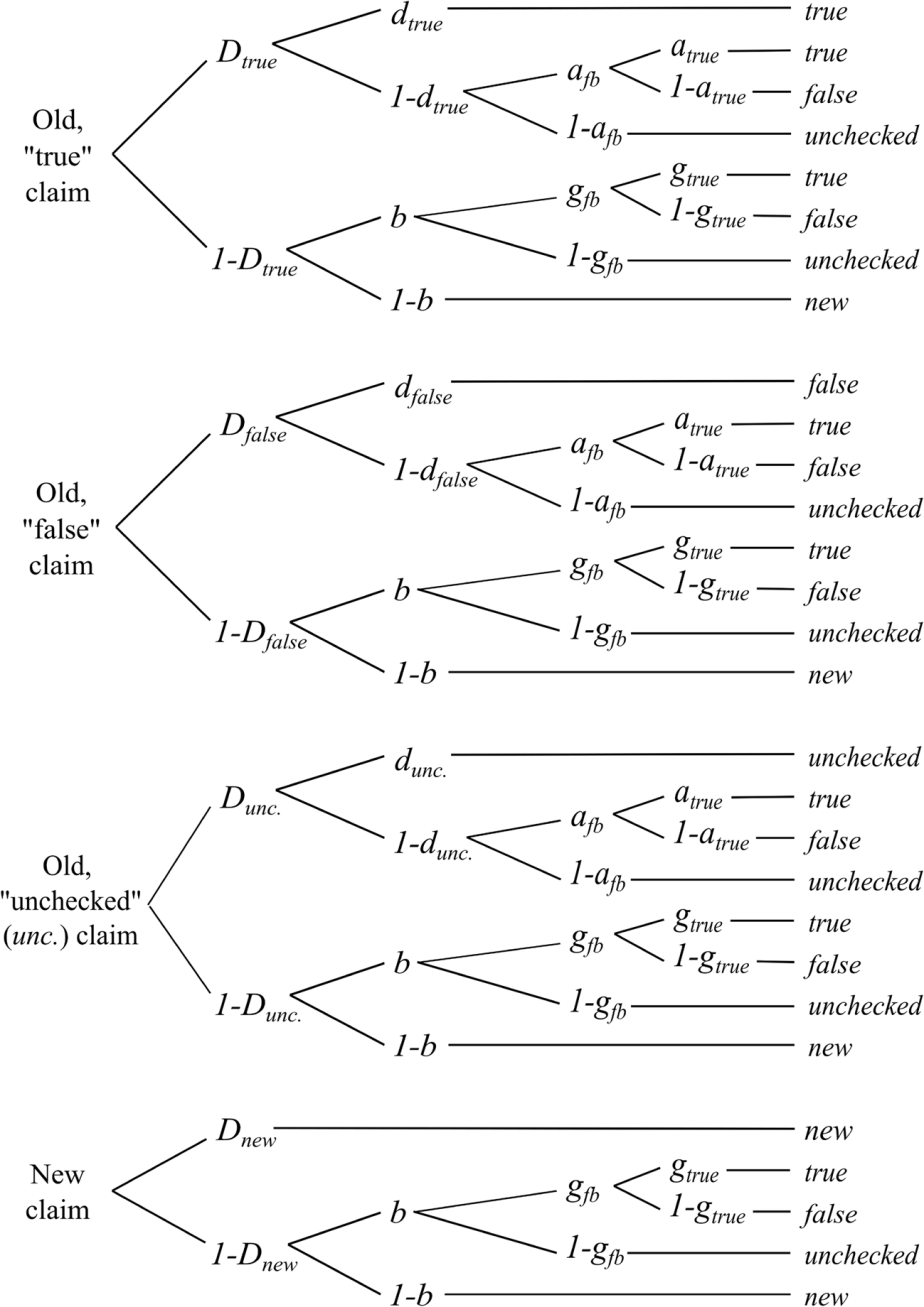
The two-high-threshold variant of the three-sources model. Each processing tree refers to a different claim condition (old “true”, old “false”, old “unchecked”, new). Each branch of a processing tree represents assumptions about the interplay of different cognitive processes underlying a particular response (true, false, unchecked, new). The model’s parameters reflect item memory (D), feedback memory (d), and different guessing processes (b, a, and g)

To explain the model’s assumptions in more detail, let us consider advertising claims that were associated with “true” feedback as an example. The model predicts that a claim that had been presented with “true” feedback is recognized as old with probability $D_{true}$ in the later memory test. If the claim is recognized, participants additionally remember the respective “true” feedback with probability $d_{true}$. If the feedback is not remembered (probability $1-d_{true}$), however, the feedback response relies on guessing processes. Specifically, participants guess with probability $a_{fb}$ that they have received veracity feedback. If so, they guess with probability $a_{true}$ that the feedback was “true” or with the complementary probability $1-a_{true}$ that the feedback was “false”. If recognition for the old, true statement fails in the first place (probability $1-D_{true}$), participants need to guess whether the statement is old (probability $b$) or new (probability $1-b$). In the case of an “old” guess, participants also need to guess whether they received veracity feedback (probability $g_{fb}$) or not (probability $1-g_{fb}$). If participants guess they have received feedback, they also have to guess whether the feedback was “true” (probability $g_{true}$) or “false” (probability $1-g_{true}$). Parallel processes are assumed for claims with “false” feedback, as well as for “unchecked” claims, and new claims. Importantly, however, the model estimates separate item- and feedback-memory parameters for true, false, and unchecked claims, allowing us to test, among other things, whether feedback memory differs between “true” and “false” feedback in the two experimental groups. By contrast, the guessing processes are the same for all types of claims regardless of their veracity or whether they have been presented before. Figure 1 shows the model’s assumptions in the form of processing trees.

### Power analysis

To determine the required sample size, we performed a power analysis for the relevant parameter comparison of the MPT model using the program multiTree (Moshagen, 2010). For this analysis, we specified a significance level of $\alpha$ = .05, a target power of 1-$\beta$ = .95, and a to-be-detected effect equal or larger than $\Delta$ = .15 between feedback-memory parameters $d_{true}$ and $d_{false}$. Assumptions about the parameter values, which are required for the power analysis, were based on the parameter estimates of a pilot study ($N = 66$).^1^ Our power analysis indicated a minimum sample size of $N = 208$ participants to reliably detect the specified effect in the $d$ parameters predicted by the Spinozan model. To detect differences in the $d$ parameters as predicted by the expectation-violation model, the estimated minimum sample was marginally lower ($N = 206$). We thus set our target sample size to $N = 208$.

## Results

### Preregistered primary analyses

#### Item memory

Equating item memory for new and unchecked statements was required to obtain an identifiable base model. The base model incorporating these minimal assumptions fit the data, $G^2$(2) = 3.17, $p$ = .205. As specified in our preregistration, we tested whether the item-memory parameters ($D$) could be further restricted to reduce the complexity of the model. However, the $\Delta G^2$-statistic (Hu & Batchelder, 1994) indicated that model fit decreased significantly when equating the item-memory parameters across base-rate groups, $\Delta G^2$(3) = 70.03, $p$ $< .001$, or across “true” and “false” feedback, $\Delta G^2$(2) = 43.30, $p$ $<.001$. Following the preregistered data-analysis plan, we therefore retained the initial base model. The parameter estimates of the model are displayed in Table 1.

#### Feedback memory

To test whether memory is equivalent for “true” and “false” feedback (as predicted by the Cartesian model), better for “false” than for “true” feedback (as predicted by the Spinozan model), or better for whatever information is unexpected in the given context (as predicted by the expectation-violation model), we compared the base model to a model assuming equivalent memory for “true” and “false” feedback in each base-rate group. If the model fit does not significantly decrease due to the restrictions, this speaks in favor of the Cartesian model and against both the Spinozan model and the expectation-violation model. In contrast, if the restrictions significantly decreased the model fit, this would indicate that memory differs between “true” and “false” feedback, as predicted by both the Spinozan model and the expectation-violation model. Despite the large sample size, $d_{true}$ and $d_{false}$ did not significantly differ within groups, $\Delta G^2$(2) = 2.27, $p$ = .321, which provides evidence in favor of the Cartesian model and against the Spinozan model and the expectation-violation model. As specified in the preregistration, this result made any further tests of the specific predictions that were derived from these competing models redundant.^2^

**Table 1:** Item-memory parameters *D* and feedback-memory parameters *d* with 95% confidence intervals for each base-rate group and feedback type

	Item memory			Feedback memory		
Group	$D_{true}$	$D_{false}$	$D_{unchecked}$	$d_{true}$	$d_{false}$	$d_{unchecked}$
HTB	.53 [.50,.55]	.64 [.60,.67]	.52 [.49,.55]	.62 [.57,.67]	.66 [.61,.71]	.26 [.16,.36]
HFB	.55 [.52,.59]	.47 [.45,.50]	.46 [.43,.49]	.61 [.55,.67]	.57 [.49,.66]	.36 [.29,.44]

*Note.* HTB = high ’true’ base-rate; HFB = high ’false’ base-rate

#### Guessing

Similar to the tested restrictions on the item-memory parameters, we also aimed to constrain the guessing parameters as much as possible. However, all preregistered constraints significantly reduced model fit (constraint 1: $b_{HTB} = b_{HFB}$, $\Delta G^2$(1) = 7.88, $p$ = .005, constraint 2: $a_{fb} = g_{fb}$ and $a_{true} = g_{true}$ in each group, $\Delta G^2$(4) = 19.46, $p$ = $< .001$, constraint 3: $a_{fb, HTB} = a_{fb, HFB}$ and $g_{fb, HTB} = g_{fb, HFB}$, $\Delta G^2$(2) = 27.51, $p$ $< .001$). Following our preregistered data-analysis plan, we therefore kept the initial base model. Guessing-parameter estimates are displayed in Table 2.

**Table 2:** Guessing parameters with 95% confidence intervals for each base-rate group

	Guess ’old’	Guess ’feedback’		Guess ’true’	
Group	*b*	$a_{fb}$	$g_{fb}$	$a_{true}$	$g_{true}$
HTB	.25 [.23,.27]	.57 [.52,.63]	.76 [.71,.80]	.66 [.60,.72]	.73 [.68,.79]
HFB	.29 [.27,.32]	.75 [.70,.81]	.77 [.74,.81]	.26 [.20,.32]	.28 [.23,.32]

*Note.* HTB = high ’true’ base-rate; HFB = high ’false’ base-rate

There is empirical evidence that people’s contingency-based or schema-based expectations affect their guessing behavior in a memory test (e.g., Arnold et al., 2013; Bayen & Kuhlmann, 2011; Street & Kingstone, 2016). The different expectations about the truth or falsity of the presented advertisement claims should thus be reflected in the parameters that indicate the probability of guessing “true”. Specifically, values for $a_{true}$ or $g_{true}$ above .50 would indicate a guessing tendency in favor of “true” feedback, and values below .50 would indicate a guessing tendency in favor of “false” feedback. In the HTB group, $a_{true}$ and $g_{true}$ were both significantly larger than .50, $\Delta G^2$s (1) $\ge$ 24.93, $p$s $< .001$, confirming a tendency towards guessing “true”. In contrast, both parameters were significantly smaller than .50 in the HFB group, $\Delta G^2$s(1) $\ge$ 50.09, $p$s $< .001$, confirming a tendency toward guessing “false”. These results indicate that the base-rate manipulation in the study phase had worked as intended.

### Additional analyses

Further analyses, described in detail in an online supplement on the OSF (see https://osf.io/xn84u/), yielded the following results: First, an exploratory analysis of the item-memory parameter $D$ revealed that participants showed significantly better item memory when the feedback violated participants’ expectations about the veracity of a claim. Second, following our preregistered data-analysis plan, the model-based analysis was supplemented with an analysis of the directly observable behavior using the Conditional Feedback Identification Measure (CFIM), calculated as the proportion of correct feedback attributions for correct “old” responses (Murnane & Bayen, 1996). The CFIM did not differ between “true” and “false” feedback, but performance was better for both “true” and “false” feedback than for the unchecked condition. There was no group effect but a significant interaction, showing increased attribution to the expected feedback type. This pattern is consistent with the guessing bias identified in the model-based analysis. Hence, the observed pattern of correct feedback attributions did not reflect participants’ feedback memory but their context-informed guesses. This finding aligns well with the *informed* Cartesian account of Street and Kingstone (2016), which takes context-informed guesses into account. Third, in line with our preregistration, participants’ scores on the skepticism-towards-advertising scale were compared between base-rate groups. Advertising skepticism was significantly higher in the HFB group than in the HTB group, providing additional evidence that the base-rate manipulation affected participants’ expectations about the veracity of advertising claims.

## Discussion

Across various contexts in everyday life, people do not only encounter truthful information but are also confronted with false information. It is therefore important to understand how people process and remember information about truth and falsity. In previous experiments on this issue (e.g., Nadarevic & Erdfelder, 2013; 2019), participants encountered as much true as false information. In everyday life, however, the rates of true and false information can differ across contexts. In many contexts, when reading newspapers or when talking with colleagues, friends, or relatives, people may expect that most of what is communicated is truthful, consistent with “tacit assumptions underlying the conduct of conversations in daily life” (Skurnik et al., 2005, p. 723). In some contexts, for example in digital advertising and social media (Kozyreva et al., 2020), people may have reason to expect most information to be false. It is thus interesting to examine whether the expected base rates determine the tagging of truth and falsity.

In the present study, three models were contrasted. The Cartesian model implies that people tag both truth and falsity. The Spinozan model implies that only falsity is tagged. While both models predict the tagging process to be invariant to the base rates of truth and falsity, the expectation-violation model implies that the tagging of falsity is favored in contexts of expected truth and the tagging of truth is favored in contexts of expected falsity. A fair test of this assumption requires successfully manipulating participants’ expectations. Therefore, two manipulation checks were conducted to assess the success of the base-rate manipulation. First, we compared guessing probabilities for “true” and “false” feedback bet-ween base-rate groups. Second, we compared participants’ skepticism towards advertising between groups. Both tests confirmed the effectiveness of the base-rate manipulation in shaping participants’ expectations. However, irrespective of the base rates of alleged true and false information in the study phase, participants remembered the truth and falsity of advertising claims equally well. This finding supports the Cartesian model and provides evidence against both the Spinozan model and the expectation-violation model.

The support of the Cartesian model is consistent with most evidence on veracity tagging in which memory was separated from guessing (Nadarevic & Erdfelder, 2013, 2019). The present results extend these previous findings by demonstrating that the Cartesian model holds, both when truth and when falsity is expected. Note, however, that we only considered context-based expectations. Hence, the present results do not address whether prior knowledge and statement plausibility affect truth tagging. As yet, studies on this matter have revealed mixed results (Niedziałkowska & Nieznański, 2021; Vorms et al., 2022).

While most previous studies used trivia statements (e.g., “*Manama is the capital of Bahrain.”*), the present study supports the Cartesian model using advertising statements (e.g.,”*Gaton jam consists of fruits cooked directly without additives.”*), thereby showing consistent results across statement types. The consistency across statements is not trivial, considering Nadarevic and Erdfelder’s (2019) finding that memory was equivalent for the truth and falsity of trivia statements, but better for the truth than for the falsity of Hopi statements. Hopi statements, supposedly translations of a foreign language spoken by a group of Native Americans (e.g., “A *monishna is a star.”*, see Gilbert et al., 1990), may be special because they are informative only when being true (Hasson et al., 2005). For example, knowing that *a monishna is NOT a star* is completely pointless. For trivia statements or advertising statements, in contrast, ”false” feedback can be as informative as “true” feedback. For example, knowing that *Sydney is NOT Australia’s capital* or knowing that *a certain washing powder is NOT particularly eco-friendly* can be as informative as comparable affirmative statements. Therefore, the falsity of a statement may be worth remembering, just as the truth of a statement. Overall, there might be a prioritization of veracity tags that are particularly informative considering the statement’s content, supporting a Cartesian model with optional rather than mandatory tagging (see Nadarevic & Erdfelder, 2019).

In summary, the results of our preregistered experiment suggest that memory for truth and falsity is best predicted by the Cartesian model, in line with previous work (Ford & Nieznański, 2023; Nadarevic & Erdfelder, 2013, 2019). Going beyond previous work, our experiment demonstrates that the Cartesian model generalizes to advertising statements, irrespective of the base rate of truth and falsity.

## Footnotes

1 More detailed information on the pilot study and the power analysis are available on the OSF: https://osf.io/z4fwb/

2 A reviewer suggested an even more rigorous test of the Spinozan model would imply omitting the $d_{true}$ and the $d_{unchecked}$ parameter form the MPT model (as no veracity tags should be added to statements presented as true or unchecked). We implemented this test, which was not part of our preregistered analysis plan, by setting $d_{true}$ and $d_{unchecked}$ to 0. This restriction, which is equivalent to the suggested model without a $d_{true}$ and $d_{unchecked}$ parameter, resulted in a significant model misfit, $\Delta G^2(4) = 847.76, p < .001$, implying that the strong hypothesis that only false statements are tagged can be rejected as it is incompatible with the data.

## Declarations

### Open Practice Statement

The data, analysis code, and materials for the experiment are available at the Open Science Framework (https://osf.io/x963s/) and the experiment was preregistered (see https://osf.io/x963s/registrations).
